# Understanding COVID-19 Halal Vaccination Discourse on Facebook and Twitter Using Aspect-Based Sentiment Analysis and Text Emotion Analysis

**DOI:** 10.3390/ijerph19106269

**Published:** 2022-05-21

**Authors:** Ali Feizollah, Nor Badrul Anuar, Riyadh Mehdi, Ahmad Firdaus, Ainin Sulaiman

**Affiliations:** 1 Universiti Malaya Halal Research Centre, Universiti Malaya, Kuala Lumpur 50603, Malaysia; ainins@um.edu.my; 2 Department of Computer System & Technology, Faculty of Computer Science & Information Technology, Universiti Malaya, Kuala Lumpur 50603, Malaysia; badrul@um.edu.my; 3 Department of Information Technology, College of Engineering and Information Technology, Ajman University, Ajman P.O. Box 346, United Arab Emirates; r.mehdi@ajman.ac.ae; 4 Faculty of Computer Systems and Software Engineering, Universiti Malaysia Pahang, Gambang, Kuantan 26300, Malaysia; firdausza@ump.edu.my

**Keywords:** vaccine, halal vaccine, COVID-19, social media, Twitter, Facebook, sentiment analysis, emotion analysis

## Abstract

The COVID-19 pandemic introduced unprecedented challenges for people and governments. Vaccines are an available solution to this pandemic. Recipients of the vaccines are of different ages, gender, and religion. Muslims follow specific Islamic guidelines that prohibit them from taking a vaccine with certain ingredients. This study aims at analyzing Facebook and Twitter data to understand the discourse related to halal vaccines using aspect-based sentiment analysis and text emotion analysis. We searched for the term “halal vaccine” and limited the timeline to the period between 1 January 2020, and 30 April 2021, and collected 6037 tweets and 3918 Facebook posts. We performed data preprocessing on tweets and Facebook posts and built the Latent Dirichlet Allocation (LDA) model to identify topics. Calculating the sentiment analysis for each topic was the next step. Finally, this study further investigates emotions in the data using the National Research Council of Canada Emotion Lexicon. Our analysis identified four topics in each of the Twitter dataset and Facebook dataset. Two topics of “COVID-19 vaccine” and “halal vaccine” are shared between the two datasets. The other two topics in tweets are “halal certificate” and “must halal”, while “sinovac vaccine” and “ulema council” are two other topics in the Facebook dataset. The sentiment analysis shows that the sentiment toward halal vaccine is mostly neutral in Twitter data, whereas it is positive in Facebook data. The emotion analysis indicates that trust is the most present emotion among the top three emotions in both datasets, followed by anticipation and fear.

## 1. Introduction

The COVID-19 pandemic has introduced unprecedented challenges in the world. The health crisis has driven governments to take extraordinary measures to save lives, including lockdowns and social distancing measures. However, the combined effect of these measures and the spread of the virus resulted in an unparalleled dramatic decline in economic activity, as the affected sectors were essentially shut down [1]. Aside from economic challenges, healthcare workers are at risk of developing anxiety-related symptoms common in catastrophic circumstances [2,3]. Examples include post-traumatic stress conditions, burnout syndrome, physical and emotional exhaustion, depersonalization disorder, and dissociation [3]. Although there is no definite cure, the vaccine is the best way to fight the pandemic. It usually takes many years to develop a vaccine against a disease, and the process involves animal testing as well as multiple human trials to measure the effectiveness. For example, the typhoid vaccine took 34 years to be developed [4]. However, in the case of COVID-19, the advances of science played a crucial role in speeding up the vaccine development process. When most people are immune to an infectious disease through vaccination, this provides indirect protection, referred to as herd immunity, to those not immune to the disease [5]. With the help of vaccination, it is possible to eradicate some diseases worldwide entirely, as was the case with smallpox [5].

Vaccine recipients have different nationalities and religions. Among them, Muslims, Hindus, and Jews have questioned the content of COVID-19 vaccines [6]. Muslims are obliged to consume only halal products. Vaccines must follow halal standards to be used in Islamic countries [7]. For example, it is not allowed to use pork gelatin in vaccines, which is known to be used as a stabilizer for safe storage and transportation of the vaccines. Ebrahim [8] has explained that from an Islamic point of view, Islam is not against vaccines; however, medical actions must fulfill any one of the higher purposes of Sharia law (maqāsid al-shariah) to be considered ethical. In this context, he argues that vaccination programs may contribute to preserving national wealth (hifz al-māl) as they are a cost-effective measure to prevent disease rather than treat its symptoms. As a result, governments and religious leaders in Muslim-majority countries are talking with vaccine manufacturers, investigating production processes, and issuing directives to ensure that concerns about products prohibited by Islam do not hinder COVID-19 inoculation [7]. Pfizer, Moderna, and AstraZeneca have already declared that their COVID-19 vaccines do not contain pig products [9].

The coronavirus pandemic of 2020 is the first global health crisis in the age of social media. During this pandemic, numerous social media platforms are used to alleviate and spread harm. During epidemics, people need accurate information to modify their behavior and protect themselves, their families, and their communities against infection [10]. An infodemic can be more challenging to manage when health information messages and facts are incorporated into political narratives and online commentary that is not grounded in verified facts and evidence [10]. Despite these hazards, Gottlieb and Dyer [11] have identified several key benefits of social media during national crises. For example, social media can facilitate the distribution of new information to providers on the frontlines. The deployment of social media can expedite public health response. For example, during the massive community-wide quarantine in China, it was essential to use social media wisely to provide a channel that communicates the reasons for the quarantine and provides reassurance and practical advice to preempt rumors and panic [12].

There are research works on COVID-19 vaccines, focusing on analyzing government performance in the pandemic, vaccine certification, and acceptability of the vaccines [13,14]. However, this is the first study that uses aspect-based sentiment and emotion analyses on social media, Twitter, and Facebook data.

### Similar Works

Natural Language Processing (NLP) is a branch of machine learning dealing with text data which uses algorithms to process and analyze text. Among various branches of NLP, sentiment analysis studies text data to determine polarity of text as positive, neutral, and negative. Emotion recognition is another branch of NLP dealing with extraction and recognition of emotion in the text data [15]. It is conducted according to emotion models such as the Ekman [16], Plutchik [17], and Lovheim [18] models.

Among many research works published in sentiment analysis and emotion recognition, Deng et al. [19] proposed a deep learning method for emotion recognition using an attention-based approach to represent the correlations between text data and emotions. In another work, Deng et al. [20] proposed a method that helped designers and developers consider the emotions expressed in music and their effects on listeners’ emotions. The authors suggested a use case for their method in which a music recommender system predicted the change in emotional state of the user and recommended music based on their current emotional state. Bekmanova et al. [21] analyzed sound data from online distance learning classes to identify sentiment and emotions of students attending the classes. The recognition model was proposed based on transcription of audio data. Papapicco et al. [22] published a study in which they monitored and analyzed the emotional side of an interview. They collected data by selecting 11 participants and recording their interview. They then analyzed the recordings and extracted the emotions expressed in the interview.

There have been research works on COVID-19 vaccines. Wardhana [23] has researched the connection between halal and vaccines in research papers indexed in the Scopus website as secondary data. There were 46 occurrences of co-authorship and 68 instances of co-occurrence of authors’ topics. The results showed that the topic of the vaccine is connected to halal and fatwa or ulema council statements. Based on the keywords, vaccines and COVID-19 were a topic. The author concluded that halal and vaccines intertwine, especially in Muslim countries [23]. Hussain et al. [24] designed and implemented an artificial-intelligence-based model to analyze public sentiments on social media in the United Kingdom and the United States toward COVID-19 vaccines. They did not focus on halal discussion of the vaccines. They used data from Facebook posts and tweets posted in English in the United Kingdom and the United States from 1 March 2020, to 22 November 2020. They developed a hierarchical hybrid ensemble-based AI model for thematic sentiment analysis using a deep learning model. They reported that overall averaged positive, negative, and neutral sentiments were at 58%, 22%, and 17% in the United Kingdom, compared to 56%, 24%, and 18% in the United States. Regarding COVID-19 vaccines, they observed stronger sentiments on Twitter for the United States, with positive and negative sentiments displaying stronger increasing and decreasing trends, respectively, compared to the United Kingdom.

There are other studies conducted specifically on the halal COVID-19 vaccine from various perspectives. For example, Susilo et al. [14] examined the Indonesian Ministry of Health posts on Instagram from a political science perspective. They analyzed the posts to evaluate the performance of the ministry in overcoming hoaxes against vaccination. In another work, Sholeh and Helmi [25] obtained data from library resources to study the permissibility of the halal COVID-19 vaccine from Islamic law’s perspective. Wong et al. [13] evaluated the acceptance of COVID-19 vaccines among Muslims in Malaysia. They used internet-based surveys to collect data. The majority expressed infinite intent in obtaining the vaccine followed by probable intent.

There is a gap in the literature to explore the content of social media and evaluate the sentiment and emotion of the users towards the halal vaccine. This helps to facilitate the immunization program by alleviating users’ concerns about the vaccine. Therefore, this paper aims to understand the online discussion of halal COVID-19 vaccines and identify people’s perceptions of them. To the best of our knowledge, this is the first study to conduct aspect-based sentiment and emotion analyses of Facebook and Twitter data to find out what people think and feel about the halal COVID-19 vaccine.

## 2. Methodology

**Figure 1:**
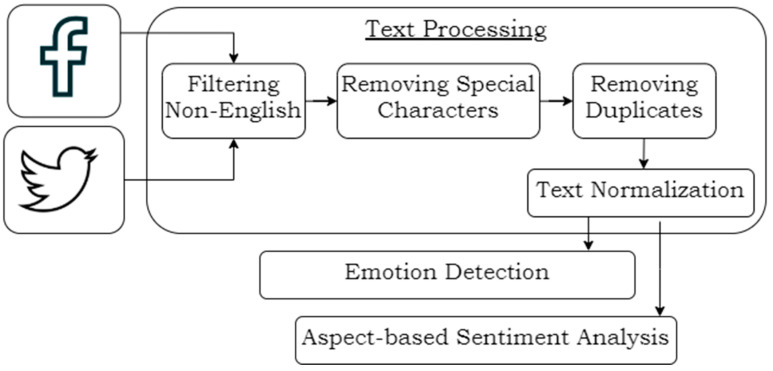
Overview of the methodology.

This section explains the methodology used in this work. Figure 1 shows the methodology in a diagram.

### 2.1. Data Collection

The first step is to collect data from Twitter and Facebook. This subsection discusses the data collection from Twitter and Facebook and the preliminary analysis of the collected data.

#### 2.1.1. Collection, Preparation, and Analysis of Twitter Data

**Figure 2:**
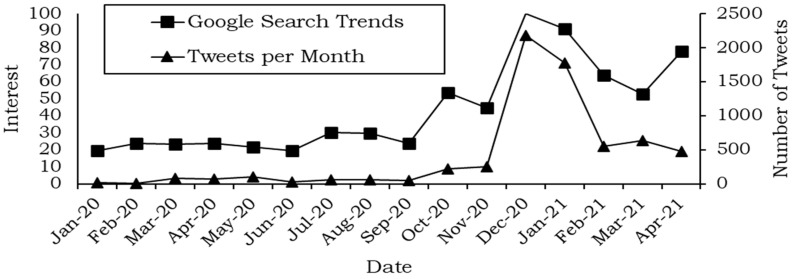
Number of tweets vs. Google Trends for halal vaccine.

Twitter provides an official Application Programming Interface (API) to retrieve tweets. However, the API only returns tweets for the last seven days. Furthermore, accessing the premium API is costly. Therefore, we used the Twint package [26] in the Python language, which utilizes the search function of Twitter to retrieve tweets on a specific topic. Tweets are collected through a Python script or directly through the command-line interface in Windows or macOS. Using Twint, we searched for the term “halal vaccine” and limited the timeline between 1, January, 2020 and 30, April, 2021. Figure 2 shows the number of tweets in the period above. In addition, we looked up the trend thanks to Google Trends [27], which shows interest in a topic over time in Google services such as search, YouTube, etc.

Figure 2 shows that in the specified timeframe, the interest identified by Google is similar to the number of tweets. Numbers between 0 and 100 represent interest over time. Zero means no interest and 100 represents maximum interest over the entire period. Therefore, interest at other dates is measured accordingly.

The search returned 6037 tweets containing both “halal” and “vaccine”. To complement each tweet, we retrieved metadata such as the time and date of creation, username, name, place, language, mention, number of replies, number of retweets, and number of likes.

Since the focus of this work is on English-language tweets, we filtered out non-English-language tweets by checking the language metadata of each tweet. An analysis of the language of the tweets revealed that 80.5% are in English, followed by Indonesian, Hindi, and Tagalog.

One of the metadata of tweets is hashtags. A hashtag starts with the hash sign (#), followed by a keyword or topic, without spaces. It has been used to mark and categorize tweets. Table 1 lists the top 10 hashtags found in the tweets and the number of likes and retweets for each hashtag.

As we can see in Table 1, the top hashtags are the expected ones, such as “halal”, “COVID”, and “COVID-19”. The presence of “Indonesia” is intriguing, showing that the world’s largest Muslim population is concerned about halal vaccines. Another hashtag is “muslimsboycottcovidvaccine”, which suggests that some users call for boycotting vaccines because their halal status is unknown. 

This hashtag was used on 9 December 2020, in the following tweet:

“In the name of the pious Shari’a, I appeal to all my Muslim believers to not take the COVID vaccine as it is not Halal certified. Moreover, no Maulana was associated with its development. #MuslimsBoycottCovidVaccine”.

Soon afterward, the hashtag started to trend, and on 11 December 2020 alone, it was featured in 46 tweets. The trend then declined. It is worth noting that this tweet is the third most retweeted tweet in our dataset, with 889 retweets and 3582 likes.

Location metadata are valuable for our analysis; unfortunately, many fields were left empty, and some tweets had an invalid location. Therefore, we decided not to use this metadata for analysis.

The tweets were then put through text processing. Each tweet contains special characters such as @ and #, links to a web page, and punctuation marks. We first converted the tweets to lowercase, and then removed any special characters, punctuation marks, and links. The clean data were also checked for duplicates. In the case of duplicate tweets, only one instance of the tweet was kept.

#### 2.1.2. Collection, Preparation, and Analysis of Facebook Data

The Facebook data were collected from CrowdTangle [28]. CrowdTangle is a Facebook-owned tool that tracks interactions in public content from Facebook pages and groups, verified profiles, Instagram accounts, and subreddits. They do not include paid ads unless those ads began as organic, non-paid posts that were subsequently “boosted” using Facebook’s advertising tools. They also do not incorporate activity on private accounts or posts made visible only to specific followers. The data include the contents of posts and users’ reactions to them; however, they do not contain the comments to the posts.

Similar to the Twitter data, we searched for the term “halal vaccine” and limited the timeline between 1 January 2020 and 30 April 2021. A total of 3918 posts were collected using the specified criteria.

The collected data have many metadata. For example, for each post, the following are available: page name, username, page category, number of page likes, number of page followers, post creation date, total interactions, number of likes, number of comments, number of shares, number of “love” reactions, number of “wow” reactions, number of “haha” reactions, number of “sad” reactions, number of “angry” reactions, number of “care” reactions, and message and description of the post.

**Figure 3:**
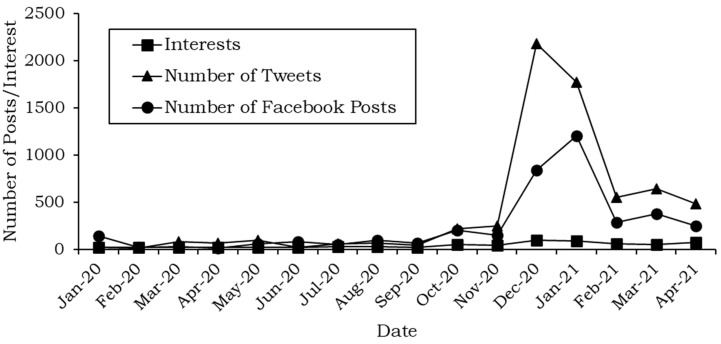
Number of Facebook posts in the collected data.

Figure 3 shows the number of Facebook posts along with the tweets and trends in the mentioned period.

Figure 3 shows that the Facebook posts followed the same pattern as the tweets and Google Trends. Between November 2020 and February 2021, the number of relevant Facebook posts and tweets is highest. This corresponds to the beginning of the vaccine’s distribution when the public sought to ascertain the halal status of the vaccine.

Some of the metadata retrieved from Facebook concern page category, that is, whether a page belongs to a person, an organization, or a group. Table 2 shows the top 10-page categories in the dataset.

News companies and agencies dominate the top 10 categories in the number of posts. The person category, which refers to pages that belong to individuals, is ranked fifth. This shows that news and government agencies are very active on Facebook. We can also analyze page categories based on the number of comments, shares, “love”, “likes”, “wow”, “haha”, “sad”, “angry”, and “care” they receive, as shown in Table 3.

As shown in Table 3, organizations and agencies are at the top of the list, receiving a high number of comments, shares, and “love”. We also see that these organizations are accepted and welcomed by the public, as their posts are shared by users.

The most shared post was published by the Indonesian Ministry of Health and received 50,343 likes. It addresses collaboration between the government of Indonesia (the most populous Muslim country) and the Imperial College London on COVID-19 vaccine development.

Unlike Twitter data, Facebook data do not contain metadata specifying the language of the posts. Therefore, we used a Python library, Whatthelang, to detect the language and filter out the non-English-language posts. Furthermore, text processing was performed on the Facebook data, including removing duplicated posts and empty posts to ensure data consistency.

Text processing was the next step. Tweets and Facebook posts contain special characters such as @ or #, punctuation marks, and links to websites. To analyze them effectively, we needed to remove those characters and clean up the text. Therefore, we filtered out non-English-language data and removed special characters and duplicated data. Additionally, we used text normalization on the data, specifically lemmatization, a method of shortening the words to their roots (for example, “eating,” “eats,” and “ate” are mapped to “eat”). This method protects the algorithms from confusion.

### 2.2. Aspect-Based Sentiment Analysis

Once the data were clean and ready for analysis, we performed aspect-based sentiment analysis. One of the most well-known research areas in computer science is sentiment analysis, which aims at extracting and identifying users’ opinions [29]. Sentiment analysis is carried out on a word, a sentence, or a whole document. Thus, it specifies the polarity of given data regardless of what they refer to. However, it is not the case in many situations [30].

**Figure 4:**
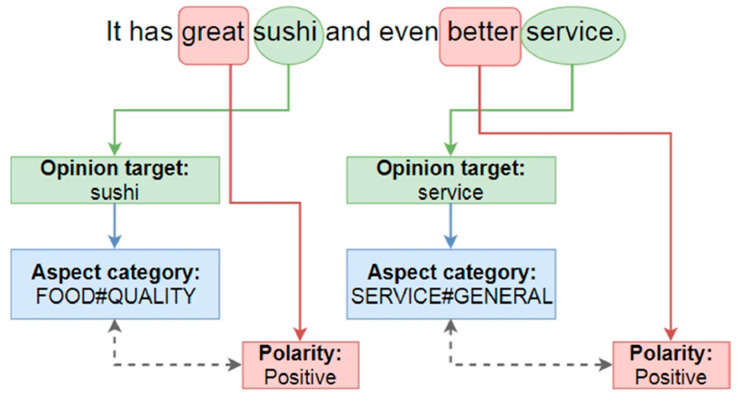
Aspect-based sentiment analysis. Reprinted with permission from Ref. [29], Copyright 2019, Elsevier.

Aspect-based sentiment analysis tries to solve this issue by identifying the topics discussed in the data and performing sentiment analysis on each [31]. Thus, it is clearer what users think about a specific topic rather than their general sentiment. Figure 4 shows the aspect-based sentiment analysis in action.

As shown in Figure 4, first, the aspect of a sentence is identified—in this case, food and service. Then, for each aspect, the polarity of the corresponding sentence is extracted, which is positive in this figure.

This approach has been used in many contexts in the literature, such as for movie reviews [32], product reviews [33], and social media analysis [34]. Therefore, we use the Latent Dirichlet Allocation (LDA) algorithm for topic identification and the VADER library [35] in Python for sentiment analysis.

The VADER library is a lexicon and rule-based sentiment analysis tool specifically tuned for sentiments expressed on social media. Since it is a pretrained library, it does not require training. It is constructed from a generalizable, valence-based, human-curated gold-standard sentiment lexicon. It is a fast library that can be used for a large volume of data, such as social media posts. The VADER library produces a score between −1 and +1, with −1 negative, +1 positive, and 0 neutral. A score greater than 0.05 is considered positive and less than −0.05 is considered negative [35]. Research such as [36] has used VADER as a Python library for sentiment analysis.

### 2.3. Text Emotion Analysis

Text emotion analysis is the process of detecting emotions in data. There are two approaches to detect emotion in text, word-based and learning-based approaches [37]. In the word-based approach, words have a predefined emotion gathered through crowdsourcing, specified based on their role in a sentence. In the learning-based approach, an algorithm is trained on labeled data. Then, it is used on new data to identify the emotion. The former approach is ready to use on the data, while the latter requires training an algorithm on a large dataset to achieve high accuracy [37]. 

NRCLex is a tool that uses the word-based approach. It is based on the lexicon dictionary of approximately 27,000 words, Canada’s National Research Council (NRC) [38], and the NLTK library’s WordNet synonym sets. Thus, it covers an extensive dictionary. It is fast for large text, and it is easy to understand [39]. It detects eight emotions in text: fear, trust, anger, anticipation, joy, surprise, sadness, and disgust. The emotions are based on Plutchik’s wheel of emotions, a famous method in psychology [17]. We chose this tool due to its extensive dictionary and fast results. Furthermore, it has been used in literature for text emotion analysis [40].

Text2emotion [41] library in Python is another word-based approach that detects a text’s anger, fear, happiness, sadness, and surprise. It is relatively fast, and the results are easy to understand. It starts by extracting the grammatical role of each word, and then specifies adjectives and adverbs, if present. Finally, the library compares the adjectives with the pretrained database and specifies the emotion associated with the word.

IBM Tone Analyzer is another word-based tool. It was developed by IBM and is accessible through API. It can detect document emotion and sentence emotion. However, the results are not easy to understand, and it is slow for large text. It also relies on an active network connection, which slows down the process.

## 3. Analysis Results

This section presents the results of the analyses carried out according to the method described in the previous section, including aspect-based sentiment analysis and text emotion analysis.

### 3.1. Results of the Aspect-Based Sentiment Analysis

We introduce the results for Twitter and Facebook data separately. This allows us to compare the two popular social media platforms.

#### 3.1.1. Aspect-Based Sentiment Analysis in Twitter Data

**Figure 5:**
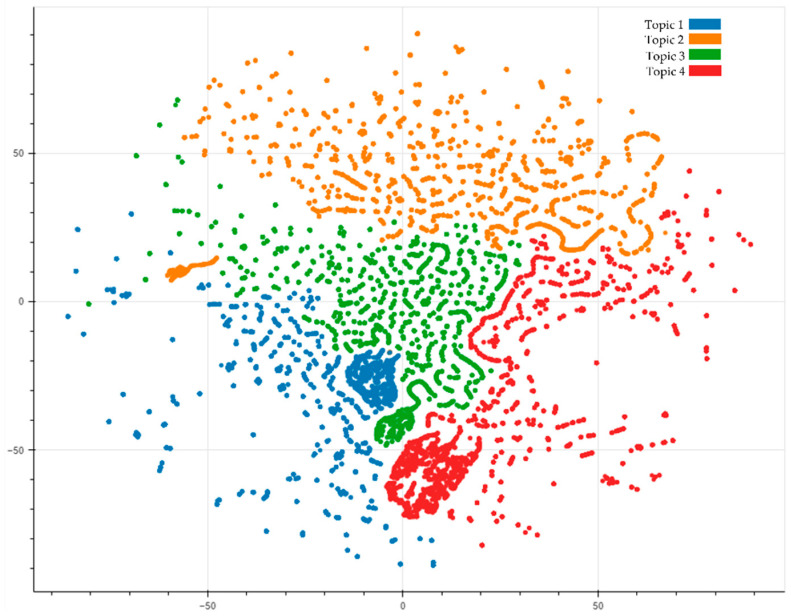
Visualization of the topic modeling analysis for Twitter data.

The cleaned tweets were used to run the LDA algorithm, which required us to specify the number of topics. We first ran the algorithm with seven topics. However, the results were vague, consisting of “halal status,” “amid halal,” “halal await,” “vaccine halal,” “halal vaccine,” “filled cow,” and “experimental vaccine”. Therefore, we tried the experiment with four topics, which resulted in meaningful topics. For visualization purposes, we plotted the dataset to see the separation of the topics. This separation shows that the dataset can be divided into individual topics and any possible overlap in the data. Figure 5 shows the results for four topics.

**Figure 6:**
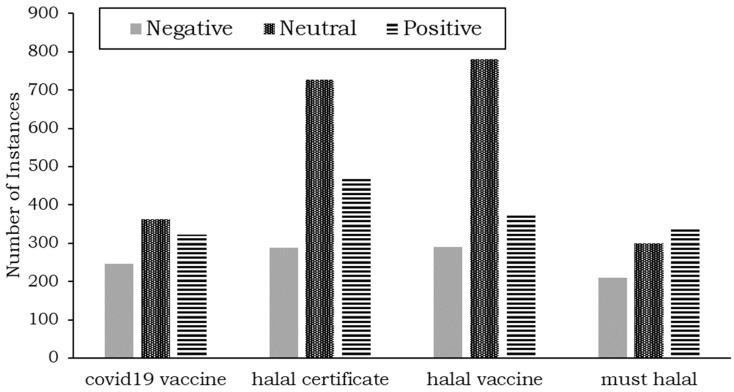
Aspect-based sentiment analysis results for Twitter data.

Figure 5 shows four topics in the Twitter dataset. A color represents each topic. We can see that the words are grouped into topics and form a cluster. We then label each topic and calculate sentiment analysis of each topic, which is shown in Figure 6.

Based on Figure 6, the “COVID-19 vaccine,” “halal certificate,” and “halal vaccine” topics are associated with neutral sentiments more than with positive or negative ones. This shows that the users are still unsure about these topics since they are relatively new. Additionally, after neutral sentiments, positive sentiments are more prevalent than negative ones for all three topics. Specifically, the difference between positive and negative sentiments is more significant for the topic of the halal certificate than for other topics. This shows that although most users have a neutral opinion about this topic, many welcome the concept of a halal certificate for the vaccine.

#### 3.1.2. Aspect-Based Sentiment Analysis in Facebook Posts

**Figure 7:**
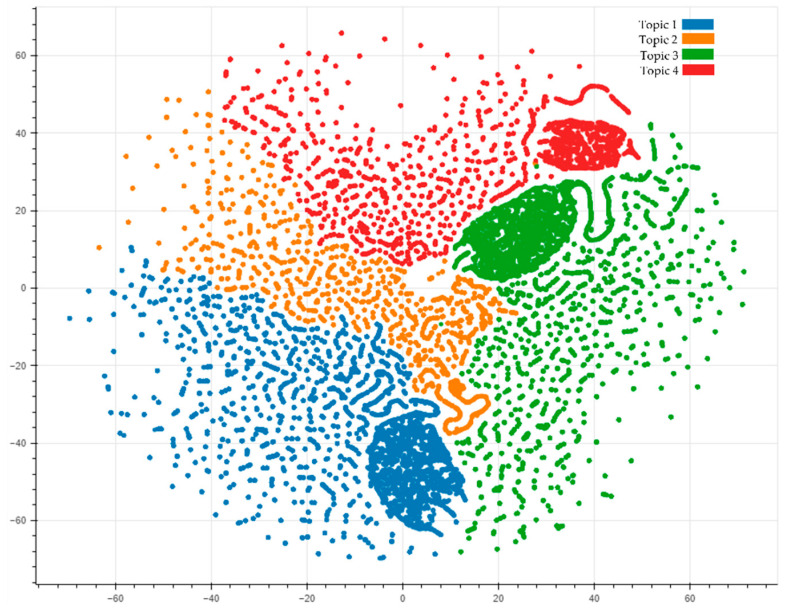
Visualization of the topic modeling analysis for Facebook posts.

Similar to the Twitter data analysis, the Facebook posts were fed to the LDA algorithm, and the topics visualization is shown in Figure 7.

**Figure 8:**
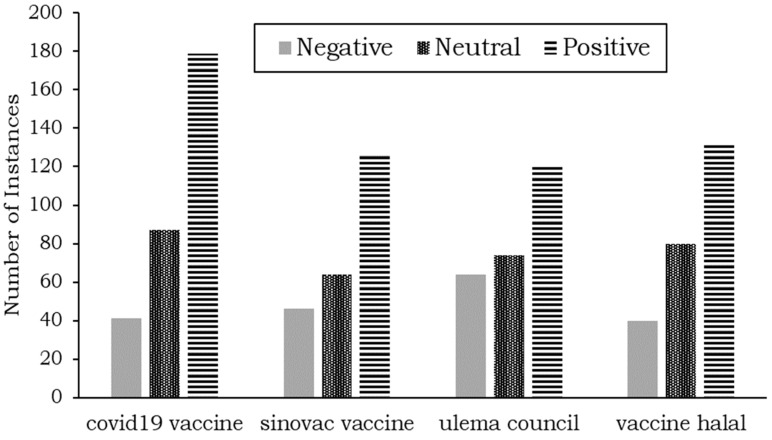
Aspect-based sentiment analysis results for Facebook posts.

Like Twitter data, the four topics are separated individually. The topics can then be labeled, and the sentiment of each topic is shown in Figure 8.

As shown in Figure 8, the topics are “COVID-19 vaccine,” “Sinovac vaccine,” “ulema council,” and “vaccine halal”. All topics show that positive sentiments are more prevalent than neutral and negative sentiments.

Regarding the ulema councils (the council that provides halal certification for products such as foods, cosmetics, pharmaceuticals, and clothing), the difference between negative and neutral sentiments is marginal. Similarly, the difference between negative and neutral sentiments is small for the Sinovac vaccine topic.

### 3.2. Results of Text Emotion Analysis

As discussed in Section 2.3, text emotion analysis is the process of specifying emotion in a given text. This section presents the results of emotion analysis for Twitter and Facebook data. Similar to aspect-based sentiment analysis, we calculate emotions for each topic, which enables granulated analysis.

#### 3.2.1. Results of Text Emotion Analysis for Twitter Data

**Figure 9:**
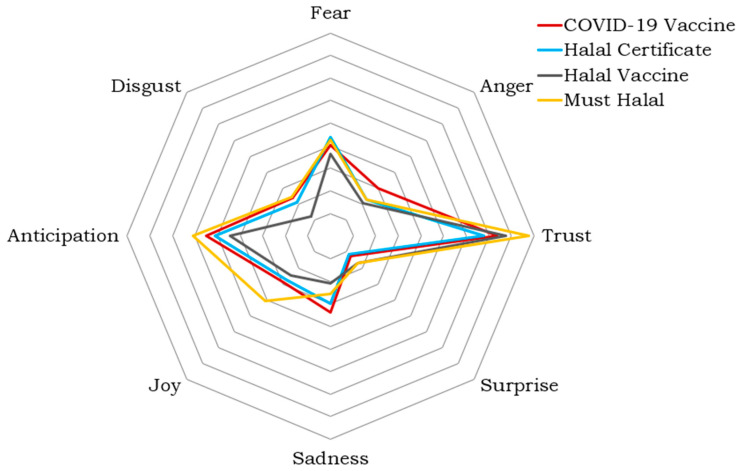
Emotion analysis for Twitter data.

The Twitter dataset, once cleaned, was analyzed using the NRCLex library in Python [37]. Figure 9 shows the results in a spider chart, which helps compare emotions of different topics in tweets. It is also easy to see the highest and lowest emotions in each topic.

Interestingly, the results show that trust is the most frequently detected emotion in the tweets. The second emotion is anticipation, followed by fear. Sadness is the fourth emotion in the “COVID-19 vaccine” and “halal certificate” topics, while joy is the fourth emotion in the other two topics.

#### 3.2.2. Results of Text Emotion Analysis for Facebook Posts

**Figure 10:**
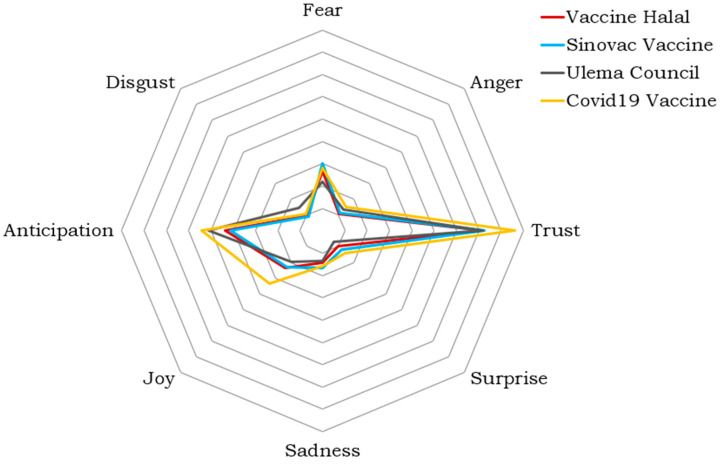
Emotion analysis for Facebook posts.

Similar to the Twitter data, the Facebook data were analyzed using NRCLex, the emotion analysis tool. Figure 10 shows the results of emotion analysis for each topic in Facebook posts.

As we can see in Figure 10, trust is the most dominant emotion, followed by anticipation. Fear is in the third rank. We can also see the sadness emotion in the results. It is ranked fourth after the fear emotion. Surprise, anger, and disgust are present in the figure with the lowest values. 

**Figure 11:**
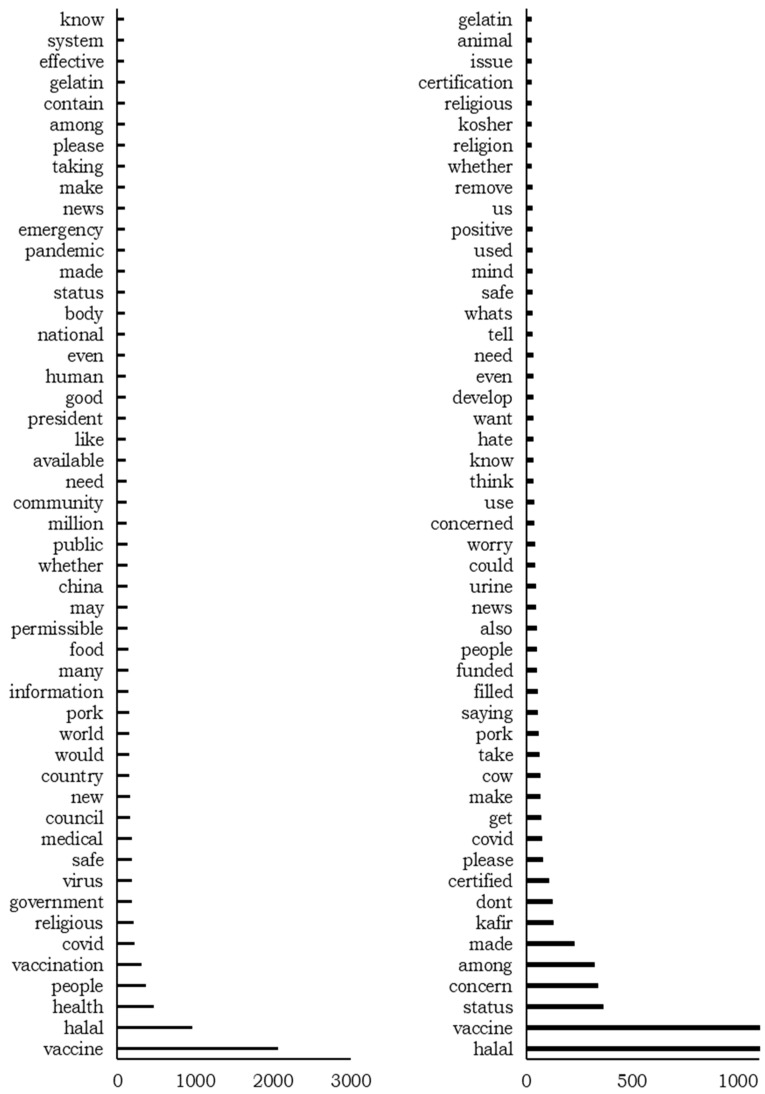
Top 50 most frequent words in Facebook (**left**) and Twitter (**right**) data for fear.

We further investigated fear as the most present negative emotion in Twitter and Facebook data. The data labeled “fear” were identified and the 50 most used words were extracted to determine what users talk about in their posts. Figure 11 depicts the top 50 words.

Figure 11 shows that in Facebook posts, users are most concerned about the religious aspect of the vaccines and whether they are halal. Specifically, they mention “pork” and “gelatin” and ask for more information on the contents of the vaccines. In addition, we find “government” and “president” in the top-50 list. Considering that most of the Facebook pages belong to news and government agencies, this shows the action of governments towards ensuring that the vaccines are indeed halal. In this regard, we can point to the most shared post about the Indonesian government’s collaboration with an institution in London to develop a vaccine and make sure it follows the Islamic guidelines.

Twitter data show a similar pattern but from a different perspective. The tweets contain “pork,” “urine,” “animal,” and “gelatin,” indicating Muslim concern about the contents of the vaccine, similar to the Facebook data. In addition, Twitter users ask for halal certification and emphasize the need for a valid certificate for the vaccine, as “certified” and “certification” are among the top 50 words in the data.

## 4. Discussion

### 4.1. Principal Findings and Implications

In this study, we analyzed Twitter and Facebook data related to halal COVID-19 vaccines. The data were collected between January 2020 and May 2021. The period between November 2020 and February 2021 saw the highest number of tweets and posts. The analysis included aspect-based sentiment analysis as well as emotion analysis. The focus of this study was on English-language tweets and posts. We identified topics in the data and calculated the sentiment for each aspect (topic). This approach has the advantage of detecting sentiments related to a specific topic rather than the whole dataset. Emotion detection for each topic was also performed, complementing the sentiment analysis. It further analyzes the emotion in the text compared to positive and negative sentiments.

Our findings indicate that online public discourse on Twitter and Facebook regarding halal vaccines reflects a genuine concern in the Muslim community. Users discussed topics such as “halal vaccine,” “halal certificate,” “must halal,” “Sinovac vaccine,” and “ulema council”. The sentiment towards these topics was somewhat similar. While users on Twitter mostly expressed neutral sentiments, the Facebook data show mostly positive sentiments. This study extends the current literature by diving deeper into the two most used social media. Researchers can expand this study by including other social media platforms and exploring the content more comprehensively. 

Since most Facebook posts were published by government and news agencies, we speculate that they tried to convey positive sentiments to users to encourage them to take the vaccine. In addition, the tweets came from the public and government agencies, contributing to the more neutral sentiment. Considering that fear is among the top emotions expressed by users, policy makers, organizations, and government agencies can use the results of this study to communicate more effectively with the public. This includes alleviating their concerns regarding the halal vaccine and assuring them that they collaborate with the respective companies to develop vaccines complying with halal conditions. 

### 4.2. Comparison with Prior Works

The results of this study corroborate those of an earlier study by Zhang et al. [42], which analyzed sentiments among the population on Facebook and Twitter. Their research showed that the older male population displayed a more significant proportion of fear and depression. In a similar work, Hussain et al. [24] analyzed public attitudes toward the COVID-19 vaccine on Facebook and Twitter. They found that the overall sentiment was positive. However, they noted that sentiments appeared more negative on Twitter than on Facebook. These results appear consistent with this study as we have positive sentiment in Facebook data and neutral sentiment in the tweets data. Lyu et al. [43] studied tweets to identify vaccine-related discussion. Their sentiment analysis showed a positive trend in general. The emotion analysis showed that trust was the most predominant emotion, followed by anticipation, fear, sadness, etc. In another similar work, Kwok et al. [44] analyzed English-language tweets from Australian users regarding the COVID-19 vaccine to extract topics and sentiments. They found that nearly two-thirds of the sentiments of all tweets expressed a positive public opinion about the COVID-19 vaccine. Among the eight basic emotions, trust and anticipation were the two prominent positive emotions observed in the tweets, while fear was the top negative emotion. The results presented in this study corroborate those of the works mentioned above.

### 4.3. Limitations

Our study focuses on analyzing social media data. The population using social media is relatively young [45], and the opinion of others is not taken into consideration. Previous studies mentioned that most social media users are 25–40 years old [46]. Collecting older generations’ opinions requires modification to the methodology or using conventional methods such as a questionnaire.

Additionally, the geolocation of the users may add bias to the data. We can focus our study on a specific region or country and analyze their opinion on the halal vaccine. Selecting a region or a country also helps to consider the culture of that region or country.

We collected data from Twitter and Facebook due to their popularity and ubiquity. Other popular social media platforms include Instagram, a platform popular for sharing pictures and videos. Although pictures and videos are different from text, the comment section of the posts is a great source of data. Reddit is also another social media site that is popular among some users.

This study filtered and analyzed English content, which leaves out other languages such as Arabic. This is due to limitations in algorithms and the fact that they are well-tuned for the English language. Researchers may analyze contents in other languages in the future when the algorithms are more robust on non-English content.

## 5. Conclusions

This study analyzed Facebook and Twitter data regarding halal COVID-19 vaccines between January 2020 and May 2021. The results showed that users discussed topics such as “halal vaccine,” “Sinovac vaccine,” “ulema council,” and “must halal”. The sentiment expressed in the data was mostly neutral, followed by positive sentiments. We investigated further by detecting emotions in each topic. We found that trust was the most present emotion, followed by anticipation and fear. Comparison between this study and similar studies corroborates our findings. Our findings may help the relevant Muslim organizations and councils make more decisive announcements regarding the halal status of the vaccines. Additionally, the collaboration between government agencies and vaccine companies, on the one hand, and Muslim organizations, on the other hand, is crucial to encourage more people to choose to take a vaccine.

## Figures and Tables

**Table 1:** List of the 10 most frequent hashtags.

Hashtag	Frequency	Retweets	Likes
halal	468	560	1065
vaccine	334	7	66
COVID-19	326	749	3722
coronavirus	119	23	65
indonesia	79	0	4
covidvaccine	73	46	132
COVID	68	43	169
muslimsboycottcovidvaccine	64	911	3692
muslim	64	0	0
islam	61	0	0

**Table 2:** Number of Facebook posts for each page category.

Page Category	Number of Posts
Media news company	236
News site	235
Community	116
Activity general	113
Person	100
Government organization	98
Learning	68
Broadcasting media production	57
Topic newspaper	46
Non-profit	43

**Table 3:** Top 10 page categories with number of comments, shares, “love”, “likes”, “wow”, “haha”, “sad”, “angry”, and “care”.

Page Category	Total Comments	Total Shares	Total Love	Total Likes	Total Wow	Total Haha	Total Sad	Total Angry	Total Care
Media news company	12,148	5188	568	25,333	798	18,474	103	232	124
News site	10,422	4033	421	30,026	739	10,363	325	209	113
Activity general	8086	2790	898	21,458	238	6004	92	1605	87
Politician	3462	1071	132	8761	159	2498	94	81	52
Government organization	2737	1628	620	11,170	161	3018	77	49	19
Person	2633	2818	1624	59,335	50	472	19	36	79
Broadcasting media production	2615	426	141	19,627	65	680	73	136	137
Topic newspaper	1566	354	39	3546	83	2435	55	28	25
Digital creator	1352	131	36	2334	49	1986	28	42	11
Police station	1224	271	143	1735	18	3723	3	55	10
